# Design of Light Driven Hole Bifurcating Proteins

**DOI:** 10.1021/acscentsci.5c00803

**Published:** 2025-08-27

**Authors:** Xiao Huang, Jonathon L. Yuly, Peng Zhang, William F. DeGrado, Michael J. Therien, David N. Beratan

**Affiliations:** † Department of Chemistry, 3065 Duke University, Durham, North Carolina 27708, United States; ‡ Department of Pharmaceutical Chemistry, 8785 University of California San Francisco, San Francisco, California 94143, United States; § Department of Biochemistry, Duke University, Durham, North Carolina 27710, United States; ∥ Department of Physics, Duke University, Durham, North Carolina 27708, United States

## Abstract

Electron bifurcation reactions divide electrons from two-electron donors into high- and low-energy pools by transporting charge on spatially separated low- and high-potential electron hopping pathways. Bifurcation delivers electrons at potentials that drive downstream reactions in photosynthesis, respiration, and biocatalysis. Recent theoretical studies have described the requirements for effective ground-state electron bifurcation. The aim of this study is to design synthetic bifurcation constructs that can be driven by light. We describe a strategy to bifurcate holes (oxidizing equivalents) efficiently with light, and we present an illustrative energy landscape that could support this design. The design focuses on the electrochemical potentials and distances between cofactors. The analysis finds that hole bifurcation may be driven efficiently with light, guiding the further development of bioinspired networks that bifurcate charge and deliver the charges with prescribed electrochemical potentials.

## Graphical abstract

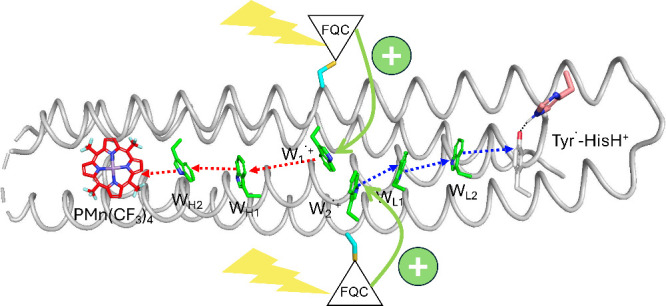

## 1 Introduction

Electron bifurcation (EB) separates electron pairs from one cofactor onto two spatially separated transport pathways. EB plays a central role in bioenergetics and biocatalysis. Examples include Complex III of mitochondria and recently discovered flavin-based electron bifurcation proteins.^1,2^ EB appears to have been discovered and exploited repeatedly by evolution.^2^ EB reactions provide strongly reducing electrons for use in some of the most challenging catalytic processes in nature, including nitrogen reduction to ammonia, carbon dioxide reduction to methane, and proton reduction to dihydrogen. EB proteins carry out these reactions with high efficiencies because the proteins can match the potentials of the bifurcating electrons to the potentials needed for the chemical transformations (i.e., the reactions occur at low overpotential).^1,3,4^

EB structures in nature perform ground-state chemistry, and the bifurcating protein is typically in contact with three redox pools: a source of two-electron cofactors (typically NADPH, NADH, or a quinone) and two spatially separated sinks for electrons, poised at high and low potential, respectively. The open-system nature of EB networks makes them difficult to replicate in the laboratory; a bioinspired mimic would require placing a bifurcating assembly into contact with otherwise electrochemically separated redox pools (thus avoiding electron short circuiting). This defines a formidable challenge.^3–6^ Beyond separating the high and low electrochemical potential pools, short circuiting through the electron bifurcating system itself must be prevented.^6^ The approach to realizing bioinspired EB structures developed here is to use light-driven electron-transfer chemistry to drive EB. An example of the free energy landscape for (ground state) EB in NADH-dependent reduced ferredoxin:NADP^+^ oxidoreductase (Nfn1) is shown in Figure 1.

**Figure 1:**
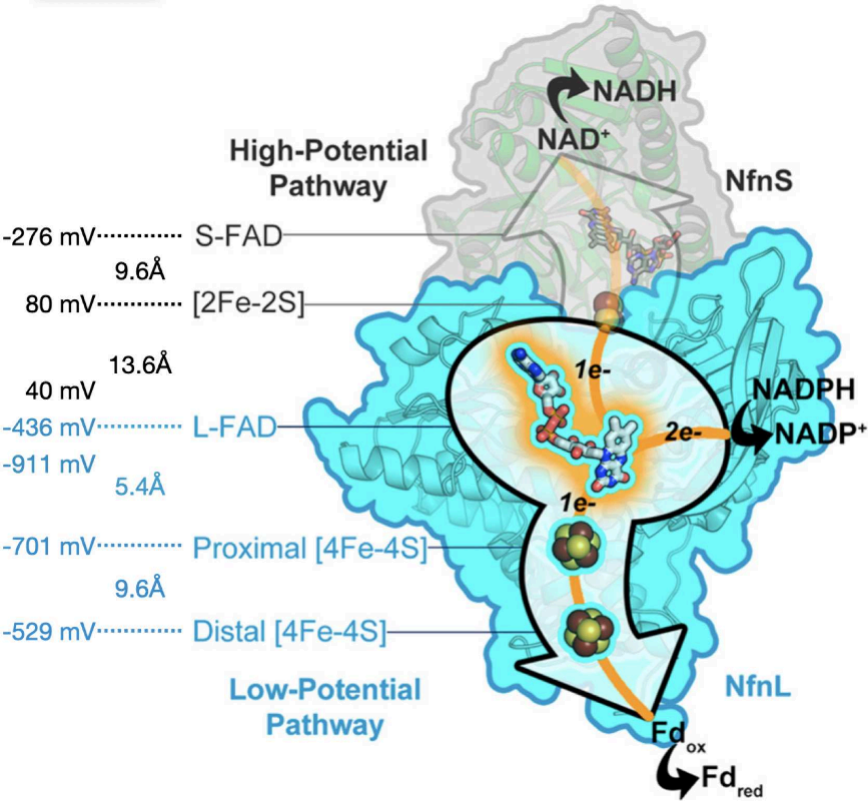
Free energy landscape of biological electron bifurcation in the Nfn1 system.^25^ The diagram illustrates the redox potentials and electron transfer pathways in the NADH-dependent reduced ferredoxin: NADP^+^ oxidoreductase (Nfn1) complex. Two electrons from a two-electron donor (NADPH) are separated into high- and low-potential branches, reducing both NAD^+^ and ferredoxin (Fd). Key cofactors, including flavins and iron–sulfur clusters, are shown with their respective redox potentials (in meV) and edge-to-edge distances (in Å) indicated along each transfer pathway. This biological system provides a reference for understanding how the free energy landscape is engineered in nature to achieve efficient electron bifurcation, which informs the design of synthetic, light-driven bifurcation constructs described in this work. This is adapted from ref 25, available under a CC-BY NC-ND license, and is copyrighted by C. E. Wise, A. E. Ledinina, D. W. Mulder, K. J. Chou, J. W. Peters, P. W. King, and C. E. Lubner.

Designing and realizing functional EB constructs is a challenging and yet unrealized objective. However, hole bifurcation (HB) offers a promising approach to realizing a charge bifurcation reaction because protein residues can be used as hole-hopping sites, and HB can be efficiently driven photochemically. Unlike conventional photochemistry, which typically generates a single high-potential oxidant, light-driven bifurcation would enable the controlled generation of two oxidizing equivalents at different potentials. This strategy allows selective channeling of photogenerated energy into two distinct physical pathways, emulating the energetic strategies found in biological systems that bifurcate electrons. Recent progress in design and synthesis allows the construction of *de novo* redox active proteins with bound cofactors, and we will used these structural scaffolds for design.^7–10^ *De novo* proteins can be designed to bind redox and photoactive cofactors.^8,9^ In the designs described here, tryptophan and tyrosine residues are used as hole acceptors.^11,12^ We hypothesize that the repulsion between two rapidly injected positively charged holes can be used to deliver one hole at a higher energy (making it more strongly oxidizing) than the other (we refer to the more strongly oxidizing hole as the “hot” hole and the less strongly oxidizing hole as the “cold” hole). Because the second hole is no longer destabilized by electrostatic interactions after the first hole leaves, it is less strongly oxidizing. This design strategy is proposed to enable the selective delivery of hot and cold holes to spatially separated hopping pathways at different potentials.

The scheme for HB in *de novo* proteins relies on two light-driven flash-quench oxidation reactions of tryptophan residues close to each other and to ruthenium chromophores bound to the protein surface (Figure 2 and Figure 3). Flash-quench chemistry has been used to study single and multistep electron transfer reactions in proteins.^13–15^ In the design of HB constructs, the Ru-chromophores are bound to the protein surface. The flash excites a bound Ru­(II) species, which features an E^0/+^ potential exceeding 2.0 V (vs NHE), which is oxidized by a freely diffusing Ru­(III) species that reside in solution. The bound Ru­(III) species then oxidizes a nearby Trp. In rapid succession, a second tryptophan oxidation is triggered by a second flash-quench reaction. The second oxidation event is designed to occur rapidly after the initial oxidation, ensuring that the first oxidized Trp remains in close spatial proximity and retains the Trp^•+^ state before deprotonation, thereby facilitating a controlled bifurcation of charge. The protein-bound Ru species return to their initial reduced state after they carry out the Trp or Tyr oxidation. The Trp radical cation state is not sufficiently oxidizing to oxidize the protein-bound Ru­(II) species in its ground state.

**Figure 2:**
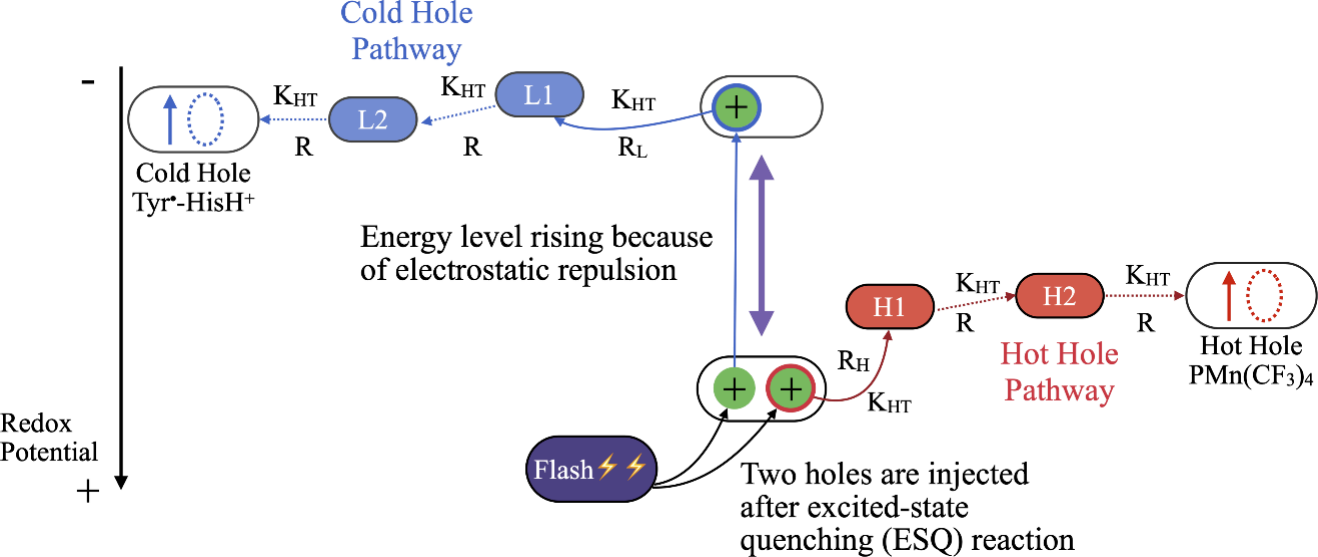
A schematic representation of a light-driven *de novo* hole bifurcating system is shown. Two flashes, in rapid succession, oxidize nearby Trp residues using photogenerated Ru­(III) species attached to the protein (via a flash-quench scheme).^26^ The photogenerated holes hop on “hot” and “cold” hole transport pathways. The holes arrive at the termini of the pathways. The redox cycle is completed when the initial electron quencher rereduces these terminal species.^26^

**Figure 3:**
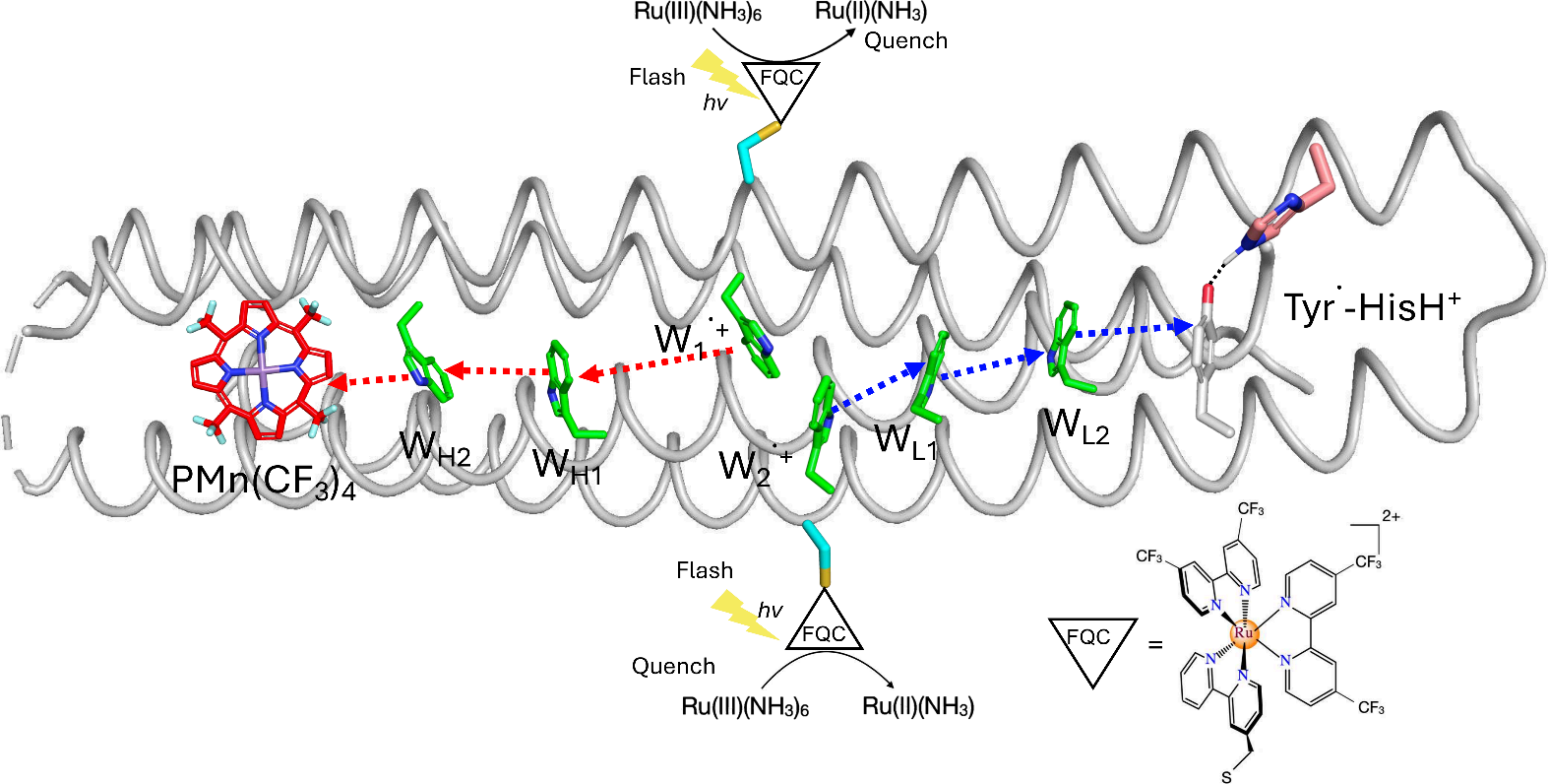
An illustrative molecular model of a designed *de novo* hole bifurcating protein. Two ruthenium flash-quench chromophores (FQC) are covalently bound to the two nearby Trp (W_1_ and W_2_) on the protein surface, undergoing photooxidation. Hole transfer proceeds along two spatially separated pathways: the hot-hole path (W_1_ → W_H1_ → W_H2_ → PMn ${\left({\mathrm{CF}}_{3}\right)}_{4}$ , in the red-arrow pathway) and the cold-hole path (W_2_ → W_L1_ → W_L2_ → Tyr^•^- HisH^+^, in the blue-arrow pathway). The two hole transfer routes terminate on acceptors tuned to different reduction potentials. This design demonstrates how light can initiate bifurcation in a synthetic, protein-based construct.

The two oxidized amino acids are designed to have strong electrostatic repulsion (on the eV scale) between them (with a hydrogen-to-hydrogen distance of ∼2.5–4.0 Å), and this repulsion energizes the holes. The first hole (i.e., the hot hole) will transit a hopping pathway that terminates with the hot hole acceptor. The second hole is stabilized after the first hole leaves, and is therefore less strongly oxidizing. The second hole then executes hole transfer (HT) on the cold hole transport pathway, arriving at the cold hole acceptor.

We aim to prepare a strong oxidant as the hot-hole acceptor and a weaker oxidant as the cold-hole acceptor. A type of cofactor that could serve as a hot hole acceptor is an electron-deficient perfluoroalkyl-substituted porphyrin macrocycle that binds Mn­(III). Such a cofactor leverages earlier designed proteins^8^ that bind meso-perfluoroalkyl-substituted Zn­(II) porphyrins^16–18^ and catalytically active Mn­(III) diphenylporphyrins,^9^ and would be anticipated to provide a redox center with a *E*_1/2_(Mn­(III)-OH/Mn­(III)-O^•^) > 1.3 V (vs NHE), suitable for accepting and utilizing a hot hole in a catalytic reaction. The electrochemical potential of the porphyrin can be tuned by functionalization, providing a path to tune the *E*_1/2_(Mn­(III)-OH/Mn­(III)-O^•^) value. A possible cold hole acceptor is a tyrosyl radical (Tyr^•^), stabilized by hydrogen bonding to a protonated histidine (HisH^+^). This Tyr-His cofactor motif has been found in natural electron transfer systems, where it facilitates long-range charge transport.^19–23^ The midpoint potential for the Tyr-His/Tyr^•^-HisH^+^ redox couple is estimated to be approximately 1.0 V (vs NHE). This redox potential provides a thermodynamic gradient that would support hole bifurcation by enabling efficient charge separation along the cold-hole transport pathway. The cold and hot hole acceptor design may be tuned to realize a 1:1 HT quantum yield, or to establish targeted hole energies or chemistries.

The distance from the initially oxidized amino acids to their adjacent cofactors is *R*_H_ (high potential site) and *R*_L_ (low potential site). Designing the system with *R*_H_ ≪ *R*_L_ favors transfer of the first hole on the hot pathway, since electron transfer rates drop rapidly with distance.^24^

We next assess the kinetics of electron transport in the landscapes described above. We find that light-driven HB can be realized on many free-energy landscapes, and we provide kinetic and thermodynamic assessments of system performance.

## 2 System Design and Analysis

### 2.1 Flash-Quench Production of Holes

The two-phase flash-quench scheme is intended to generate holes at specific molecular locations and potentials, utilizing the photoredox (flash/quench) chemistry of ruthenium complexes. A light flash excites the bound ruthenium complex, which is oxidized by a solution Ru­(III) species. The bound Ru­(III) species then oxidizes a nearby Trp (W_1_ and W_2_ in Figure 3, as indicated in Figure 4). In our design, two Trp residues in close proximity are oxidized in rapid succession by two flash-quench cycles. The resulting cationic Trp pair exhibits electrostatic repulsion between the holes. It is essential that the first injected hole remains on the $W_{1}^{•+}$ site—without deprotonating or transferring—until the second hole is introduced. To accomplish this design goal, the second flash-quench event must occur on a time scale (ps to ns)^27,28^ that is much faster than both Trp deprotonation (which occurs on the μs scale)^29^ and single-step HT (typically μs to ms).

**Figure 4:**
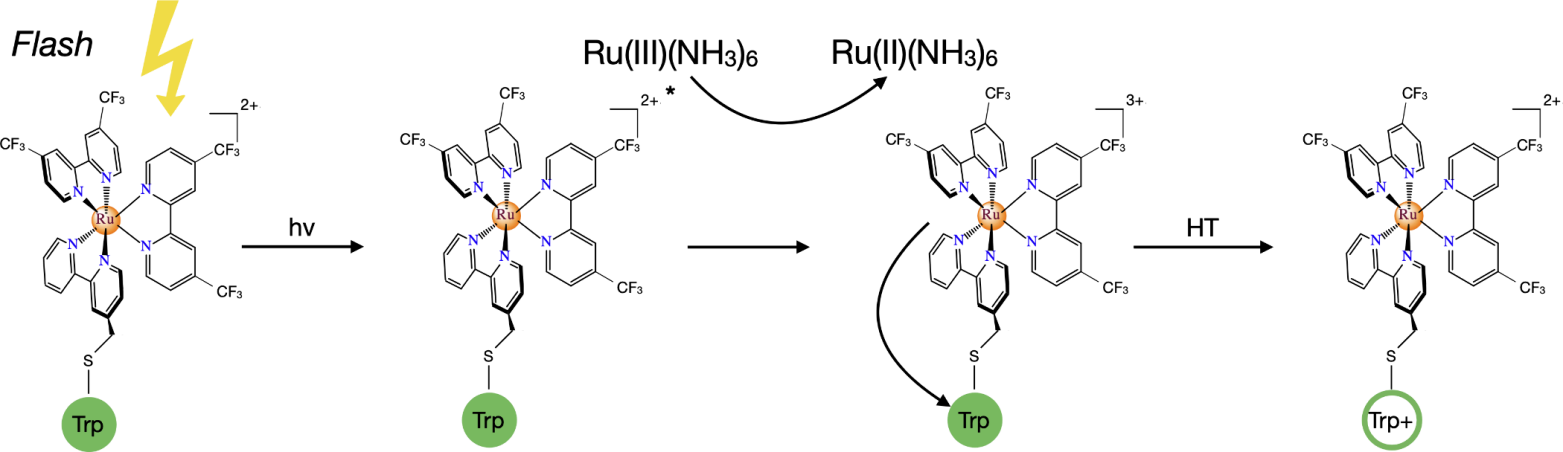
Flash quench mechanism to produce Trp radical cations.

### 2.2 Analysis of Hole Transfer Thermodynamics

#### 2.2.1 HT Driving Force

##### 2.2.1.1 Modeling Trp-Trp Interactions Using a Photolyase Model

Molecular dynamics (MD) simulations were performed on the 1DNP photolyase (PL) structure,^30^ a protein known for its multitryptophan hole-hopping pathway, to study Trp-Trp ( $W_{1}^{•+}$ and $W_{2}^{•+}$ in Figure 3) interactions. We use Trp 359 and Trp 306 to model the hole–hole interaction, since their shortest hydrogen-to-hydrogen distance is in the 2.5–4 Å range, enabling strong electrostatic interactions between holes.

MD simulations were performed on the 1DNP structure of PL using GROMACS 2024 ^31^ to model the relative positions of two nearby Trp residues (the 1DNP structure is otherwise unrelated to our bifurcating protein design). The AMBER14 force field^32^ was used on 1DNP, and the protein’s cofactors were parametrized using the GAFF2 force field.^33^ The system was solvated using GROMACS and AutoSolvate,^34^ and the TIP3P water model was used for the water molecules.^34^ The structure was energy-minimized and subsequently equilibrated in two stages: first in the NVT ensemble and then in the NPT ensemble. Each equilibration step was conducted for 2 ns at a target temperature of 300 K. The MD production run was performed for 10 ns, and the trajectory was stored. From this trajectory, 11 representative structural snapshots were taken for further analysis of the Trp dimer geometries. Fluctuations in the closest hydrogen-to-hydrogen distance, indole ring orientations, and overlap area of two Trp’s indole rings are discussed in the Results and Discussion section.

##### 2.2.1.2 Electrostatic Interactions between Trp Radical Cations

To quantify the hole–hole repulsion between the two Trp^•+^ species, MD simulations were performed on the PL (1DNP) protein. Following energy minimization and equilibration, 11 representative structural snapshots were collected from a 10 ns production trajectory. The positions of atoms in the Trp 306 and Trp 359 residues were recorded for each snapshot.

Trp 359 was capped at the N- and C-termini with hydrogen atoms. For each geometry snapshot taken for the protein with the Trp residues in their neutral state, the highest occupied molecular orbital (HOMO) energy of Trp 359 in its radical cation state (spin multiplicity = 2) was calculated using Gaussian 16.^35^ Two HOMO energy calculations were performed for the Trp 359 species with two different surrounding point charge distributions, as described below. Density functional theory (DFT) was used with the B3LYP functional and the 6-31G­(d) basis set,^36^ incorporating empirical dispersion corrections (GD3BJ)^37^ to account for weak intermolecular forces. The SCF convergence criterion (scf = xqc in the Gaussian input file) was used to ensure robust convergence of the electronic structure calculations. The surrounding protein and solvent environment (atoms within 25 Å of Trp 359 center of mass) were represented using point charges taken from the MD topology files and included in the Gaussian input file. This protocol was repeated for the 11 representative MD snapshots and approximately accounts for the dielectric screening effects of the protein.

We computed two HOMO energies for Trp 359 using two different charge states for Trp 306:

1. **Neutral Trp 306:** The atomic charges of neutral Trp 306 were extracted from the topology file generated in the MD simulations. These charges were taken from the AMBER14 force field, as implemented in the MD protein parametrization.
2. **Trp 306 Radical cation:** Instead of H atoms, Trp 306 was capped with acetyl (ACE) and methylamide (NME) groups at the N- and C-termini, respectively, to provide a model structure to calculate the restrained electrostatic potential (RESP) charge distribution for the Trp 306 radical cation state. The ACE and NME groups model the backbone of the surrounding protein, providing more accurate description of the atomic charges compared to the analysis that neglects the ACE and NME groups. The Trp 306 radical cation state was assigned a +1 charge and a single unpaired electron (charge = 1, spin multiplicity = 2). Geometry optimization for the capped Trp 306 in its radical cation state was performed using DFT with the B3LYP functional and the 6-31G­(d) basis set, using an empirical dispersion corrections (GD3BJ) to account for van der Waals interactions. The SCF convergence criterion scf = xqc was used to ensure robust convergence of the electronic wave function for the open-shell radical species.

Atomic charges for the ACE and NME capped Trp 306 species were recalculated using the restrained electrostatic potential (RESP) fitting method, which derives atomic charges by fitting to the molecular electrostatic potential obtained from quantum calculations.^38^ In this process, charge constraints were applied to the ACE and NME capping groups; their atomic charges were fixed to predefined values consistent with the AMBER14 force field. These charge values reflect the established partial charges of ACE and NME groups in the AMBER force field, ensuring compatibility with the MD simulations.

The RESP fitting process for the Trp 306 radical cation redistributed the +1 charge (other than the fixed charges on the ACE and NME groups) to the rest of the molecule. This ensures that the total charge remains consistent with the assigned +1 charge of the radical cation state, while preserving the relative ratio of the atomic charge distribution suggested by the RESP charge calculation. These steps are crucial since they can correct the inconsistency between the quantum chemical charge distribution calculation (computed for Trp 306), and the empirical force field amino acid atomic charge distribution assignment (applied to the amino acids near Trp 359, other than Trp 306). The resulting RESP charges, along with the 3D coordinates of the Trp 306 atoms were described as point charges when computing the Trp 359 HOMO energy using Gaussian 16.^35^

The electrostatic interaction energy between Trp 359 and Trp 306 was approximated as the difference in the HOMO energy of Trp 359 calculated when Trp 306 is either neutral or charged. (All other point charges remain unchanged in the calculations.) The interaction energy is

1 $$E_{\mathrm{interaction}}=E_{\mathrm{HOMO}}^{+1\mathrm{Trp}306}-E_{\mathrm{HOMO}}^{0\mathrm{Trp}306}$$

where $E_{\mathrm{HOMO}}^{+1\mathrm{Trp}306}$ is the HOMO energy of Trp 359 when Trp 306 is in the radical cation (+1 charge) state and $E_{\mathrm{HOMO}}^{0\mathrm{Trp}306}$ is the HOMO energy of Trp 359 when Trp 306 is neutral (eq 1). This approach estimates the electrostatic interaction between the two Trps, accounting for screened Coulomb interactions between the Trps as well as changes in the electronic structure induced by the charge–charge interactions between the Trp residues. This approximation neglects the structural changes that may occur when both Trp residues are charged. Our analysis of the hole repulsion between Trp residues includes only the repulsion between holes that comprise the hole bifurcating unit, namely W_1_ and W_2_ (see Figure 3).

#### 2.2.2 Hole Bifurcation Kinetics

##### 2.2.2.1 Master Equation Analysis of HB

We define the quantum yield for bifurcation as the probability that holes arrive on the high and low energy paths in a one-to-one ratio long after the flash. The HB quantum yield is computed using a master equation in a microstates framework. Each microstate corresponds to a specific hole occupancy of the cofactors in the network.

This design is motivated by the electron bifurcating protein Nfn1 (NADH-dependent reduced ferredoxin: NADP^+^ oxidoreductase), but is different in some key ways.^25^ Rather than a flavin electron bifurcation site as in Nfn1, our hole bifurcation center consists of two nearby oxidized Trp residues coupled to two spatially separated hot and cold hole transport pathways. The cold hole pathway contains two additional cofactors leading to a single cold hole trap. The hot hole pathway is constructed similarly, with two cofactors leading to a hot hole trap. The design has seven redox active modules: the bifurcating tryptophan pair, two cold hole pathway cofactors, one cold hole pathway terminus, two hot hole pathway cofactors, and one hot hole pathway terminus.

We denote each redox microstate with the vector **S**. The elements of **S** describe the occupancy of each cofactor in the system. For the cofactors [*C*_1_, *C*_2_, ..., *C*_*N*_], a general form of **S** is given by eq 2:^6^

2 $$S=\left[n_{1},n_{2},...,n_{N}\right]$$

where *n*_*i*_ is zero or +1 h (corresponding to a hole on the site).

The master equation is given by eq 3:

3 $$\frac{dP\left(S_{i},t\right)}{dt}=\underset{j}{\sum }K_{ij}P\left(S_{j},t\right)-K_{ji}P\left(S_{i},t\right)$$

where **P**(*S*_*i*_, *t*) is the probability of finding the system in microstate *S*_*i*_ at time *t*, and *K*_*ij*_ and *K*_*ji*_ are the electron transfer rates between microstates *S*_*i*_ and *S*_*j*_.

The master equation solutions are given by eq 4:

4 $$P\left(t\right)=e^{Kt}P\left(0\right)$$

where **P**_*t*_ = [*P*(*S*_1_, *t*), *P*(*S*_2_, *t*), ..., *P*(*S*_*i*_, *t*), *P*(*S*_*j*_, *t*), ...] is a vector that contains the probabilities for each microstate at time *t*; *P*(0) is the initial probability vector, and **K** is the transition rate matrix given by eq 5:

5 $$\left[\begin{array}{llll} k_{11} & k_{12} & k_{13} & \cdots \\ k_{21} & k_{22} & k_{23} & \cdots \\ k_{31} & k_{32} & k_{33} & \cdots \\ \vdots & \vdots & \vdots & \ddots \end{array}\right]$$

where *k*_*ij*_ (*i* ≠ *j*) is the transition rate from microstate ***S***_***i***_ to microstate ***S***_***j***_. The transition rates are calculated using nonadiabatic electron-transfer theory.^39,40^ The diagonal terms *k*_*ii*_ are the negative sums of all transition rates from microstate *S*_*i*_ to all other microstates *S*_*j*_, given by eq 6:

6 $$k_{ii}=-\underset{j\neq i}{\sum }k_{ji}$$

The diagonal terms described above ensure that the overall hole probability is conserved. Those diagonal terms account for probability flow out of each microstate (*S*_*i*_).

##### 2.2.2.2 HT Kinetics

We describe the nonadiabatic hole transfer rates assuming coupling both low and high frequency modes,^41,42^ given by eq 7:

7 $$k_{i\to j}=\frac{2\pi }{\hbar }<V_{ij}^{2}>\frac{1}{\sqrt{4\pi \lambda_{ij}k_{B}T}}\underset{n}{\sum }\frac{e^{-D}}{n!}D^{n}\exp\left[-\frac{{\left(\Delta G_{ij}+\lambda_{ij}+n\hbar \omega \right)}^{2}}{4\lambda_{ij}k_{B}T}\right]$$

$<V_{ij}^{2}>$ is the thermally averaged electronic coupling, λ_*ij*_ is the outer sphere reorganization energy associated with the low frequency mode, *k*_*B*_ is Boltzmann’s constant, *T* is the temperature, and $\Delta G_{ij}^{\left(0\right)}$ is the standard reaction free energy. *D* is the Huang–Rhys factor, equal to λ_in_/(ℏω), where λ_in_ is the reorganization energy of the high frequency mode. ℏω is the energy of the high frequency vibrational quantum. The mean-squared electronic coupling (eq 8) is approximated using an exponential decay model,^39^ and the parameter choices are described in the Results and Discussion section:

8 $$V_{ij}=V_{0}e^{-\beta R_{ij}}$$

where *R*_*ij*_ is the distance between the closest heavy atoms of the donor and acceptor.

### 2.3 Varying the Positions and Potentials of the Cofactors

To optimize the quantum yield for HB in our network, we used Bayesian optimization (BOp), employing the Python package bayes_opt.^43^ BOp provides a robust framework to optimize complex and computationally intensive objective functions, such as the quantum yield of the HB systems calculated through master equation analysis. The objective function (the quantum yield for hole bifurcation), *f*(**x**), depends on the design parameters **x**, including the intercofactor distances (*R*_*ij*_) and the redox potentials $\left(G_{i}^{\left(0\right)}\right)$ .

In BOp, a surrogate model uses a mathematical approximation to represent the true objective function (the quantum yield for hole bifurcation). The true objective function may be computationally costly to evaluate directly. The surrogate model provides predictions of the objective function’s behavior, based on prior observations, enabling efficient exploration of the parameter space. In our BOp model, we chose a Gaussian Process (GP) surrogate model, which offers a probabilistic framework to explore our objective function and its dependence on the HB network structure. A GP is defined by a mean function μ­(**x**) and a covariance function *k*(**x**, x*), where *k* models the correlation between different points **x** and x* in the parameter space. Here, x* refers to an unexplored point in the parameter space, where the BOp algorithm predicts the mean and uncertainty of the objective function, based on prior observations. The GP is described by (eq 9)­

9 $$f\left(x\right)∼\mathrm{GP}\left(\mu \left(x\right),k\left(x,x^{*}\right)\right)$$

The covariance function, *k*(**x**, x*), is determined by the radial basis function kernel (eq 10)­

10 $$k\left(x,x^{*}\right)=\sigma_{f}^{2}\exp\left(-\frac{∥x-x^{*}{∥}^{2}}{2l^{2}}\right)$$

where $\sigma_{f}^{2}$ is the variance, and *l* is the length scale (which determines how quickly the correlation between points decreases as the distance ∥**x** – x*∥^2^ increases). These two quantities are hyperparameters optimized using BOp. The GP predicts a mean value of μ­(**x**), which represents the expected objective function value at **x**, with a standard deviation σ­(**x**) that characterizes the uncertainty of the prediction.

BOp iteratively updates the GP model to refine the design parameters. At each iteration, the BOp algorithm evaluates the acquisition function, a mathematical criterion that guides the selection of the next set of parameters to evaluate. The acquisition function balances exploration (searching regions with high uncertainty, represented by the standard deviation σ­(**x**)) and exploitation (refining regions of the parameter space with high predicted mean values, μ­(**x**)). In the EB network optimization context, the parameter exploration involves testing new combinations of *R*_*ij*_ and $G_{i}^{\left(0\right)}$ to identify configurations that enhance the quantum yield, while exploitation refines the performance of configurations (some combinations of *R*_*ij*_ and $G_{i}^{\left(0\right)}$ ) which are already identified as high-yield candidates. For example, exploration might expand the search for more possible *R*_*ij*_ values that optimize quantum yield, while exploitation fine-tunes $G_{i}^{\left(0\right)}$ for cofactors parameter combination designs that were already identified as promising. The upper confidence bound (UCB) acquisition function is commonly used to balance this trade-off. It is defined in eq 11 as

11 α(x)=μ(x)+κσ(x)

where κ is a tunable parameter that controls the trade-off between exploration and exploitation. A larger κ value prioritizes exploration, sampling regions with high uncertainty, while a smaller κ value emphasizes exploitation in the parameter search, refining regions with high predicted performance.

BOp proceeds as follows. First, the GP is constructed using the observed data points (here, generated on HB networks with randomly chosen site potentials and nearest-neighbor distances) (**X**, **y**), where **X** contains sampled parameter combinations and **y** contains their corresponding objective function values. The a posteriori predictive distribution of the objective function at a new point x* is then calculated. Here, x* represents a candidate point for evaluation, and its predicted mean and variance are given in eqs 12 and 13 by

12 $$\mu \left(x^{*}\right)=k^{T}{\left(K+\sigma_{n}^{2}I\right)}^{-1}y$$

13 $$\sigma^{2}\left(x^{*}\right)=k\left(x^{*},x^{*}\right)-k^{T}{\left(K+\sigma_{n}^{2}I\right)}^{-1}k$$

The covariance matrix **K** quantifies the relationship between the two random variables: the input parameters **x** (e.g., *R*_*ij*_ and $G_{i}^{\left(0\right)}$ ) and the corresponding objective function values **y** (e.g., quantum yield). **k** is the covariance vector between the observed points and the new point x*, *k*(x*, x*) is the covariance of the new point with itself (equal to the prior variance at x*), and $\sigma_{n}^{2}$ represents the noise variance in the observations (assumed to be 0 in our study). The acquisition function α­(**x**) is evaluated across the parameter space by systematically predicting the mean and uncertainty of the objective function at potential candidate points **x**. The next point **x**_next_ (eq 14) is selected as

14 $$x_{\mathrm{next}}=\arg\max_{x}\alpha \left(x\right)$$

The true objective function *f*(**x**_next_) is then evaluated, and the GP is updated with the new data point (**x**_next_, *f*(**x**_next_)). This process is repeated iteratively until convergence, which in this study refers to finding a parameter configuration that maximizes the quantum yield (near 100% yield).

By iteratively updating the GP and balancing exploration with exploitation, the BOp efficiently navigates the high-dimensional parameter space, identifying designs that maximize the HB quantum yield. An example of a near-100% quantum yield design is discussed in the Results and Discussion section.

## 3 Results and Discussion

We compute the HB quantum yield from the doubly oxidized Trp dimer. As described in Sect. 2.2.1, the interaction energy between the oxidized Trp residues ( $W_{1}^{•+}$ and $W_{2}^{•+}$ in Figure 3) was approximated by performing electronic structure analysis on representative snapshots based on PL. Trp 359 in PL was modeled as a radical cation, while the charge of Trp 306 was switched between its neutral and radical cation states. The HOMO energies of Trp 359 were computed for the neutral and oxidized configurations of Trp 306, including the effects of atoms within 25 Å as point charges taken from the AMBER force field. The difference between the HOMO energies for the neutral and charged Trp 306 units energizes HB. The kinetics of HB is analyzed using the master equation approach described above. Bayesian optimization^44^ was used to generate a set of HT network parameters to optimize the HB quantum yield described in above section 2.3. Parameters that are optimized with the Bayesian approach include the edge-to-edge distances among nearest-neighbor cofactors and the redox potentials of the cofactors (the fixed HT parameters appear in Table 2).

### 3.1 Repulsion between Trp Holes

The MD simulation of PL was run for 10 ns using GROMACS, with details described in Sect. 2.2.1 above. The simulated distance between nearest Trp 359 and Trp 306 atoms (including modeled hydrogen atoms added to the structure) was 3.1 Å, and the average distance between atoms was 3.5 Å. The Trp indole rings are neither stacked nor coplanar (see Figure 5 and Figure 6).

**Figure 5:**
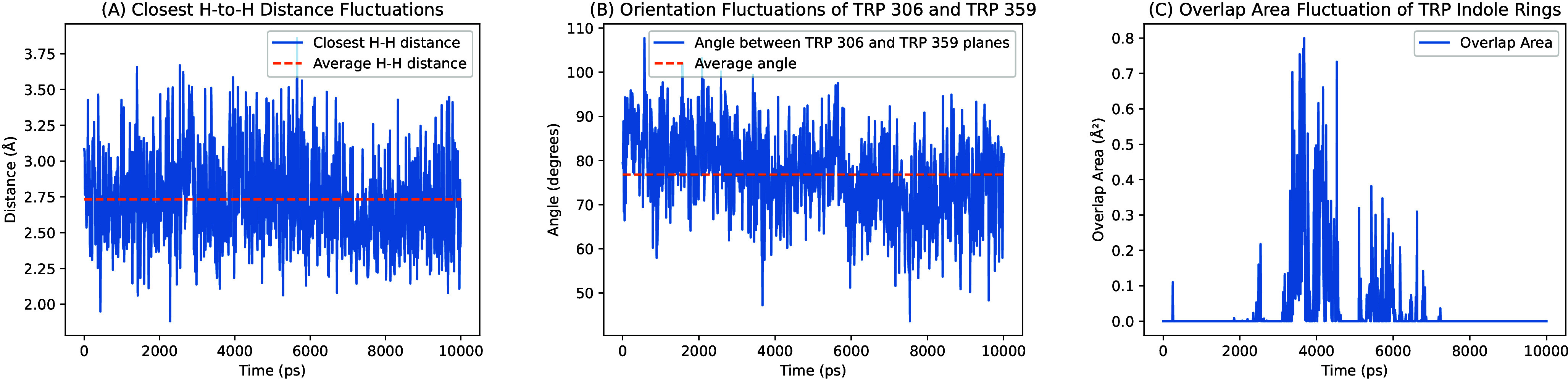
MD analysis of Trp 306 and Trp 359 in photolyase (PDB ID: 1DNP). (A) Closest hydrogen-to-hydrogen distance fluctuations between Trp 306 and Trp 359 in the molecular dynamics simulations. The closest H–H distance fluctuates between ∼2.0 Å and 3.75 Å, with an average distance of 2.75 Å. (B) Fluctuations in the angle between the planes of Trp 306 and Trp 359 during the simulations. The angle fluctuates between 50° and 110°, with an average of ∼80°. (C) The fluctuation of the overlap area between two indole rings of Trp 306 and Trp 359. Significant overlap area occurs intermittently during the trajectory, with the overlap area peaking at approximately 0.8 Å^2^. This analysis highlights the fluctuations among the Trp residues that influence hole transfer and bifurcation.

**Figure 6:**
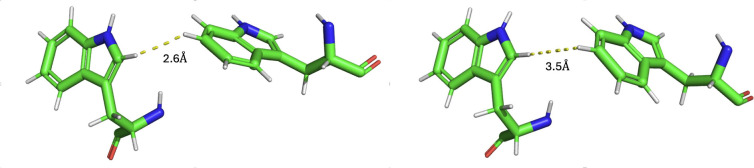
Two typical geometries of Trp 306 and Trp 359, taken from molecular dynamics simulations of PL. The closest hydrogen-to-hydrogen distance between the Trps fluctuates during the simulation (from ∼2.5 Å to 4 Å); the two indole rings are neither stacked nor coplanar in PL.

From the 11 representative Trp conformations drawn from MD simulation, two examples are shown in Figure 6. As described in Section 2.2.1, we computed the HOMO energies of the Trp 359 radical cation when Trp 306 is neutral or in the radical cation state. These values allow us to estimate the electrostatic repulsion energy between Trp radical cation states. Atomic charges (taken for the Amber force field) for all protein and water atoms within a 25 Å radius of Trp 359 were included in the quantum calculations.

The Trp radical cation interaction energy, averaged over the 11 MD-extracted conformations, was 1.6 eV (see Table 1). This repulsion energy is sufficient to drive hole bifurcation, as we describe below in our kinetic analysis.

**Table 1:** Interaction Between Bifurcating Holes on Nearby Trps

Trp dimer configuration	electrostatic repulsion energy (eV)
1 ps	1.6849
1000 ps	1.6939
2000 ps	1.7143
3000 ps	1.6074
4000 ps	1.6115
5000 ps	1.5676
6000 ps	1.5913
7000 ps	1.6618
8000 ps	1.6419
9000 ps	1.6403
10000 ps	1.5581
On Average	1.6339

### 3.2 Hole Bifurcation Kinetics

The electrochemical potential and distance parameters used in the HT kinetic analysis are indicated in Figure 8. The parameters of eq 7 and eq 8 are summarized in Table 2. These include reorganization energies, vibrational coupling constants, tunneling parameters typical of biological electron transfer processes, and the numerical convergence criteria for the calculations are also listed.

**Table 2:** Parameters Used in the HT Kinetic Analysis

parameter	value/description
Outer sphere reorganization energy (λ_outer_)	0.9 eV^45^
Inner sphere reorganization energy (λ_inner_)	0.075 eV (ℏω × *D*)
High-frequency vibrational mode energy (ℏω)	0.15 eV^39,46^
Electron-vibration coupling strength (*D*)	0.5 ^39,46^
Tunneling interaction decay constant (β)	0.6 Å^–1^ ^39,46^
Electronic coupling constant (*V*_0_)	0.01 eV^39,46^
Shortest edge-to-edge HT cofactor distance	5 Å
Numerical convergence criteria (*N*)	100 (sufficient for the sum in eq 7 to converge)

**Figure 7:**
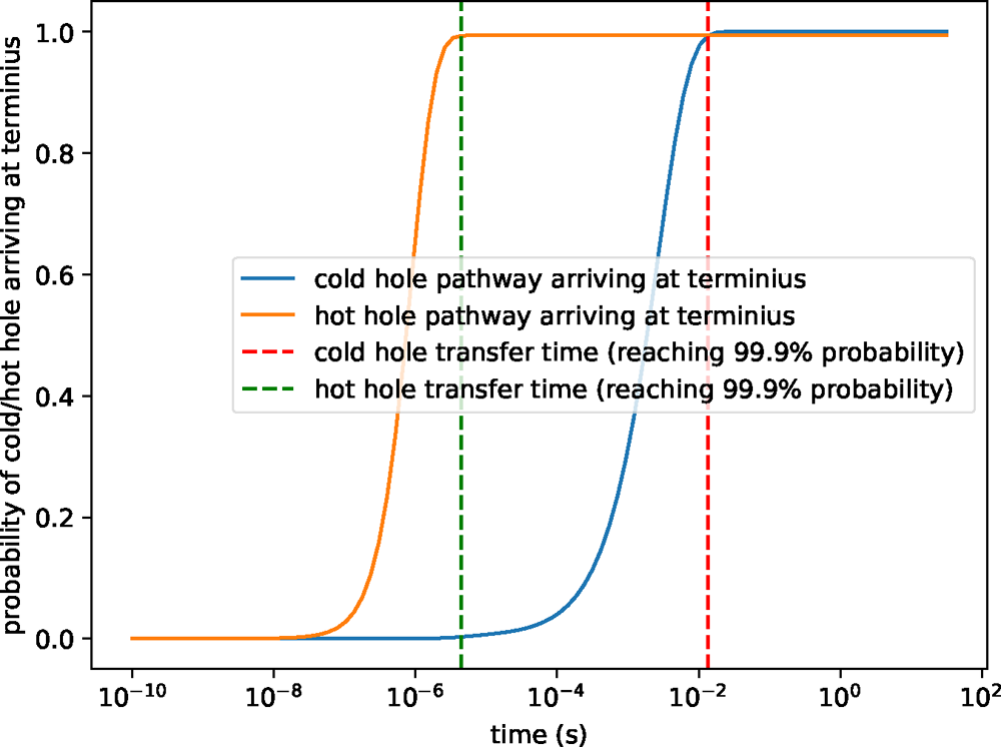
Probability of cold and hot holes arriving at their respective termini as a function of time in the designed HB system. The blue curve represents transport of the cold hole, and the orange curve represents transport of the hot hole. The red dashed line marks the estimated time for completion for cold hole transport, approximately 10^–2^ s. The green dashed line represents the estimated transfer time for the hot hole to complete transport, approximately 10^–5^ s. These time scales reflect differences in the free energy landscapes and transfer kinetics for the two pathways, with the hot-hole pathway exhibiting faster transfer because of its higher driving forces (and correspondingly lower activation free energies). These results indicate qualitatively different time scales for hot and cold hole transport in a high efficiency HT network.

**Figure 8:**
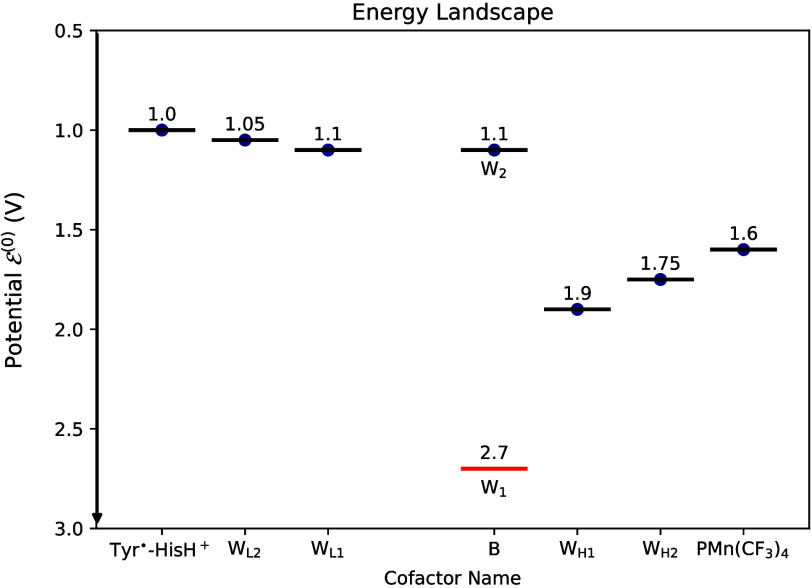
Example of an optimized HT energy landscape, indicating the relative electrochemical potentials of the cofactors (in Volts). The vertical axis indicates electrochemical potential $E^{\left(0\right)}$ . The vertical axis is plotted downward to align with the representation in Figure 2, reflecting the inverse relationship between energy levels and redox potentials. The potential of the cold-hole termini (possibly Tyr^•^- HisH^+^ in our design) defines the 1.0 V (as reference potential). The potential of the hot-hole termini (possibly PMn ${\left({\mathrm{CF}}_{3}\right)}_{4}$ in our design) is about to be 1.6 V compared to the reference potential. The cofactors that support hole hopping on both the hot- and cold-hole pathways could be Trp residues. All of the other electrochemical potential values are generated by BOp (described in Section 2.3) and aligned with reference potential. For the cofactors on the low potential (cold hole) pathway, the potentials are *W*_L1_ = 1.1 V, and *W*_L2_ = 1.05 V. For the high potential (hot hole) pathway cofactors, the potentials are *W*_H1_ = 1.9 V, and *W*_H2_ = 1.75 V. The bifurcation site, B, is a charged tryptophan (Trp+) dimer where the first Trp (*W*_1_) has potential = 2.7 V and the second Trp (*W*_2_) has potential = 1.1 V. This energy landscape was designed to produce nearly 100% HB quantum yield, with the maximum probability for bifurcation occurring to the microstate [1 0 0 0 0 0 1], corresponding to hole population of the L3 and H3 cofactors. Cofactor edge-to-edge distances are 5 Å, except for the larger 10 Å distance between W_L1_ and the bifurcation site B (driving the first hole in W_1_ onto the hot hole pathway, since it has a shorter electron transfer distance).

The hot hole (in *W*_1_) moves first from the bifurcating Trp dimer (denoted B) to the cofactors *W*_H1_, *W*_H2_, and the hot-hole pathway terminus (e.g., PMn ${\left({\mathrm{CF}}_{3}\right)}_{4}$ in our design). Then the cold hole (in *W*_2_) moves from the bifurcating Trp dimer to cofactors W_L1_, W_L2_, and the cold-hole pathway terminus (e.g., Tyr^•^- HisH^+^ in our design). The optimized distance between bifurcating site (B) and W_L1_ that is found to produce a 1:1 HT yield for the hot and cold paths is 10 Å, and the distance between B and W_H1_ is 5 Å for the energy landscape shown in Figure 8. The kinetics is computed for a time of one second. This time scale allows the bifurcation process to run to completion. (Our kinetic simulations do not include hole filling on the terminal acceptors by a sacrificial electron donor in solution, which will permit the next cycle of flash-quench HT kinetics. The hot hole could short circuit to the cold hole pathway, thus dissipating some of the stored energy.) Figure 7 shows the estimated time for hole transfer on each pathway: the cold hole pathway requires approximately 10^–2^ s to complete the transfer, while the hot hole pathway requires about 10^–5^ s. Introducing sacrificial electron donors to the solution may be used to reset the system for subsequent photocycling of the HT assembly.

The present study focuses on exploring the concept of light-driven HB, using a single illustrative network design as a proof of principle. Detailed explorations of how the structural flexibility of the *de novo* proteins may influence the cofactor orientation and reduction potentials, and thus HB performance (including short-circuiting) are beyond the scope of this study, but are targeted for future analysis.

In this study, we modeled light-driven HB in an essentially closed kinetic system (we have employed sacrificial electron donors to complete the kinetic cycle). That is, the sacrificial electron donors do not block electron short circuiting in the network. While sacrificial donors will be essential to reuse the terminal redox species and to enable continuous operation in a photochemical experiment, the theoretical analysis focuses on the fundamental bifurcation reactions. A detailed analysis of the resetting step and its kinetics will be addressed in a subsequent study.

In the earlier (open system, ground state) study, we found that only one free energy landscape produced effective EB at steady state. The free energy landscape that we described here as an example of high-efficiency light-driven HB does not map onto the universal landscape for near-reversible, steady-state, open-system EB. As such, we suspect that strongly driven HB (also, likely, EB) in closed systems can be realized on many free-energy landscapes.

### 3.3 HB Efficiency

Starting in the initial microstate [0,0,0,(1,1),0,0,0] (two holes on the bifurcating cofactors), the targeted HB microstate is [1,0,0,(0,0),0,0,1]. The master equation analysis shows nearly 100% production of this microstate 1 s after the simulation begins.

## 4 Conclusions

Using the tools of kinetic modeling of molecular simulation, we demonstrated the feasibility for realizing high-efficiency light-driven hole bifurcation in *de novo* constructs. The efficiency depends upon the judicious design of the positions and potentials of the hole transport components. We report a network design for light-driven HB that effectively bifurcates holes. The strongly driven, closed-system, HB networks explored here differ from the near-reversible open-system EB structures explored in biological EB proteins.^6,47,48^ Studies of irreversible bifurcation reactions are in their early stages.^49^ We find that the closed, strongly driven HB networks may bifurcate with high efficiency on landscapes that would not support near reversible EB in open systems.

Our analysis indicates that the hole–hole repulsion between Trp radical cations is sufficient to enable light-driven HB (1.6 eV on average), producing holes with distinctive oxidizing potential on the terminal hole acceptors. The strong hole–hole repulsion between Trp radical cations may fluctuate as the side chain positions dynamically adjust to the repulsive force. However, on average, this repulsion should remain significant and could provide a means to energize oxidizing equivalents.

This study establishes a framework to realize bioinspired *de novo* light-driven HB constructs, with promising applications in photocatalysis and energy conversion.

## △

Lewis-Sigler Institute for Integrative Genomics, Princeton University, Princeton, New Jersey 08540, United States
